# Association of IGF1 with glycemic control and occurrence of severe hypoglycemia in patients with type 1 diabetes mellitus

**DOI:** 10.1530/EC-12-0012

**Published:** 2012-06-21

**Authors:** Louise Færch, Anders Juul, Ulrik Pedersen-Bjergaard, Birger Thorsteinsson

**Affiliations:** 1 Department of Cardiology, Nephrology and Endocrinology H Hillerød University Hospital Dyrehavevej 29DK-3400, Hillerød Denmark; 2 Department of Growth and Reproduction, Rigshospitalet DK-2100, Copenhagen Denmark; 3 Faculty of Health Sciences University of Copenhagen DK-2200, Copenhagen Denmark

**Keywords:** type 1 diabetes, IGF1, hypoglycemia, HbA1c

## Abstract

**Objective:**

GH is implicated in the counter-regulatory response to hypoglycemia. We tested whether IGF1 levels are associated with occurrence of severe hypoglycemic events in patients with type 1 diabetes and whether the IGF1 concentration is influenced by glycemic control.

**Methods:**

A total of 228 outpatients with type 1 diabetes were included in a *post hoc* analysis of a 1-year observational study on severe hypoglycemia. Serum total IGF1 was measured at entry into the study. The occurrence of severe episodes of hypoglycemia, mild symptomatic, and biochemical as well as hypoglycemia awareness status was assessed. Also patients were included in a multiple regression analysis to investigate the role of HbA1c in the IGF1 concentration.

**Results:**

IGF1 levels were associated with neither severe hypoglycemia in the entire cohort (*P*=0.30) nor in any gender nor when confining the analysis to those with long-standing diabetes (>20 years) (*n*=112, *P*=0.68) and those with both long-standing diabetes and undetectable C-peptide (*n*=51, *P*=0.067). Levels of IGF1 were associated with neither mild symptomatic hypoglycemia (*P*=0.24) nor biochemical hypoglycemia (0.089) nor hypoglycemia awareness (*P*=0.16). At a multiple regression analysis, HbA1c was negatively associated with IGF1 (*P*=0.001).

**Conclusion:**

In type 1 diabetes, circulating IGF1 levels are negatively associated with glycemic control. However, IGF1 levels were not associated with occurrence of hypoglycemia or hypoglycemia awareness in these patients.

## Introduction

GH is part of the second-line counter-regulatory response to hypoglycemia and acts by promoting gluconeogenesis. In long-standing type 1 diabetes with counter-regulatory failure in terms of diminished glucagon and adrenaline responses, normal GH secretion is important to avoid progression of hypoglycemic episodes into severe hypoglycemia. GH is reflected by insulin-like growth factor 1 (IGF1), which is used to evaluate the GH axis, because GH has great diurnal variation. Total IGF1 has only limited diurnal variation (1). Patients with type 1 diabetes and GH deficiency are at increased risk of having severe hypoglycemia (2, 3, 4). Recent data suggest an association between GH axis and risk of severe hypoglycemia. Thus, low levels of circulating IGF1 are associated with increased risk of severe hypoglycemia in early pregnancy in women with type 1 diabetes (5). It is, however, not known whether this is a causal association or whether a low IGF1 concentration is a consequence of exposure to recurrent hypoglycemia. If the latter is the case, a positive association between IGF1 levels and HbA1c would be expected. Previous studies have supported the existence of a negative association between IGF1 levels and HbA1c (6, 7, 8), and one study found that the IGF1 level in patients with type 1 diabetes is low but not correlated with HbA1c levels (9).

The purpose of this study was to assess whether an association between low IGF1 and risk of severe hypoglycemia exists in a cohort of nonpregnant adult patients with type 1 diabetes and to explore whether a similar association exists with mild and biochemical hypoglycemia and with glycemic control as indicated by HbA1c levels.

## Materials and methods

The study is a *post hoc* analysis of a 1-year prospective study of the frequency of severe hypoglycemia in a cohort of 228 outpatients with type 1 diabetes (Table 1). Part of the population has previously been described (10). The study was approved by the regional ethics committee. Severe hypoglycemia was defined as an episode in which the patient needed assistance from another person to restore the blood glucose level. All such events were reported on telephone within 24 h and validated according to the triad of Whipple. Mild symptomatic hypoglycemia was defined as episodes with symptoms of hypoglycemia manageable by the patient. Biochemical hypoglycemia (blood glucose concentration <3.5 mmol/l) was assessed by monthly self-monitored five-point blood glucose profiles with measurements before the three main meals, before bedtime and at 0300 h. From these data, we calculated the percentage of hypoglycemic values. Hypoglycemia awareness was defined by a validated method based on self-reported ability to perceive hypoglycemia where those who always sense a hypo are classified as aware, those who often sense a hypo are impaired, and those who only sense a hypo occasionally or never are classified as unaware (11). HbA1c was measured spectrophotometrically (DCA-2000, Bayer; normal range 4.1–6.4%, standardized against the Diabetes Control and Complications Trial) at each visit to the outpatient clinic, and individual average values for the study period were calculated (10). Nonfasting serum total IGF1 was measured at entry into the study and determined by a solid-phase enzyme-labeled chemiluminescent immunometric assay (Immulite 2000, Diagnostic Products Corporation, Los Angeles, CA, USA). Standards were calibrated toward the WHO NIBSC IRR 87/518. The detection limit was 20 μg/l. C-peptide levels were measured by RIA (AutoDELFIA, Wallac Oy, Turku, Finland). Subjects were classified as being C-peptide negative if the value was below the detection limit of 10 pmol/l (10).

### Statistical analysis

To evaluate the association between IGF1 and severe hypoglycemia, biochemical hypoglycemia, and mild hypoglycemia we applied a log-linear negative binomial model. An association between hypoglycemia awareness and IGF1 was assessed by linear regression. *P*<0.05 (two-sided) was considered to be significant. The possible determinants of IGF1 were evaluated by multiple regression. Total IGF1 at baseline was the dependent variable with age, gender, GH, duration of diabetes, HbA1c, body mass index (BMI), C-peptide level, hypoglycemia awareness class, number of severe hypoglycemic events the previous year, and daily insulin dose as independent variables. The independent variables were chosen because of their possible influence on IGF1 (6, 8, 12, 13, 14) and their individual significant relationship with IGF1 in univariate regression analyses. Calculations were performed with SPSS software package (Version 18.0; Chicago, IL, USA).

## Results

During the study, 235 episodes of severe hypoglycemia were reported by 85 patients (37%). The HbA1c level was negatively associated with severe hypoglycemia (*P*=0.01, regression coefficient −0.77, 95% confidence interval (CI) −0.64 to −0.94), meaning that for each time HbA1c increases by 1% point the rate of severe hypoglycemic events decreases by 23%. Occurrence of severe hypoglycemia was not associated with any late diabetic complications.

### IGF1 and hypoglycemia

The average serum IGF1 level was 111 μg/l (36–479) μg/l (median (range)). As expected levels of IGF1 were associated with GH levels in univariate analysis (*P*<0.0001, regression coefficient 3.6 μg/l, 95% CI 2.0–5.2). In multiple regression analysis, total IGF1 remained associated with GH (*P*=0.004, regression coefficient 2.2 μg/l, 95% CI 0.7–3.7 μg/l).

**Figure 1:**
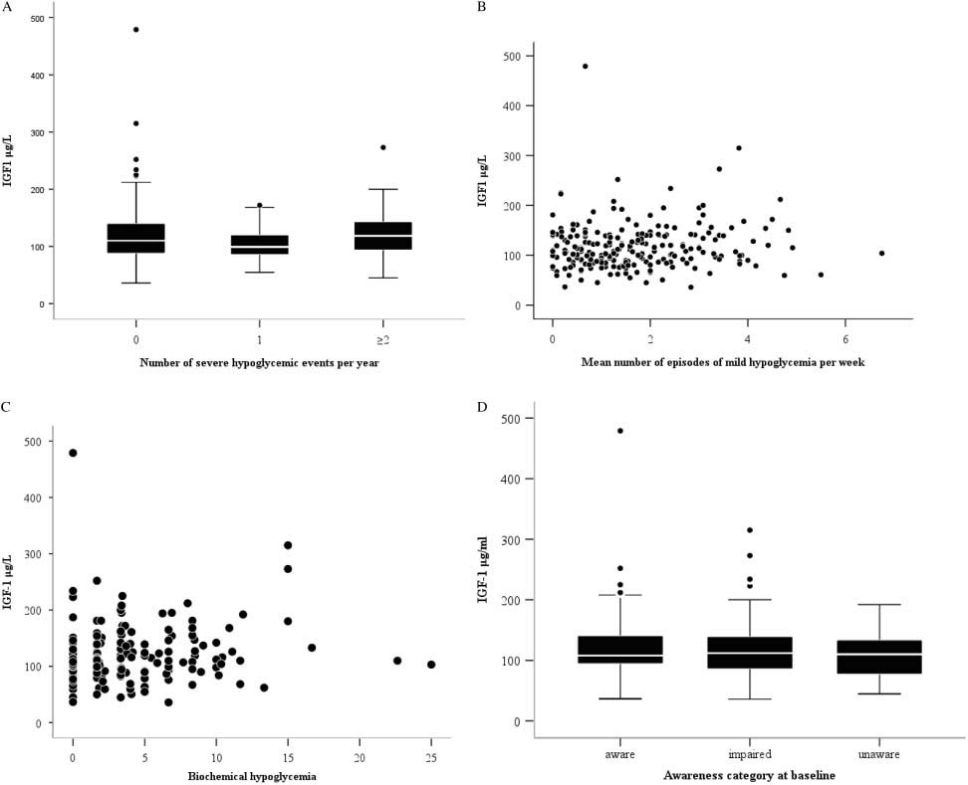
Relationship between total IGF1 concentration and frequency of (A) severe hypoglycemia (*P*=0.30), (B) mild symptomatic hypoglycemia (*P*=0.24), (C) biochemical hypoglycemia (blood glucose <3.5 mmol/l) (*P*=0.089), and (D) hypoglycemia awareness classification (*P*=0.14).

IGF1 levels were associated with the occurrence of severe hypoglycemia neither in the entire cohort (*r*=0.002, *P*=0.30; Fig. 1A) nor in any gender. Median serum IGF1 levels were 120, 106, and 123 μg/l in patients with 0 (*n*=143), 1 (*n*=41), and ≥2 (*n*=44) severe hypoglycemic events respectively. Confining the analysis to those with long-standing diabetes (>20 years) (*n*=112), who are likely to be affected by impaired glucagon and catecholamine responses, did not alter the result (*P*=0.68). When limiting the analysis to only those with long-standing diabetes as well as undetectable C-peptide (*n*=51) any differences were also absent (*P*=0.067). GH was not associated with severe hypoglycemia (*P*=0.86).

Levels of IGF1 were associated with the frequencies of neither mild symptomatic hypoglycemia (*P*=0.24) nor biochemical hypoglycemia (*P*=0.089) nor with the class of hypoglycemia awareness (*P*=0.14) (Fig. 1A, B, C and D).

### IGF1 and glycemic control

**Figure 2:**
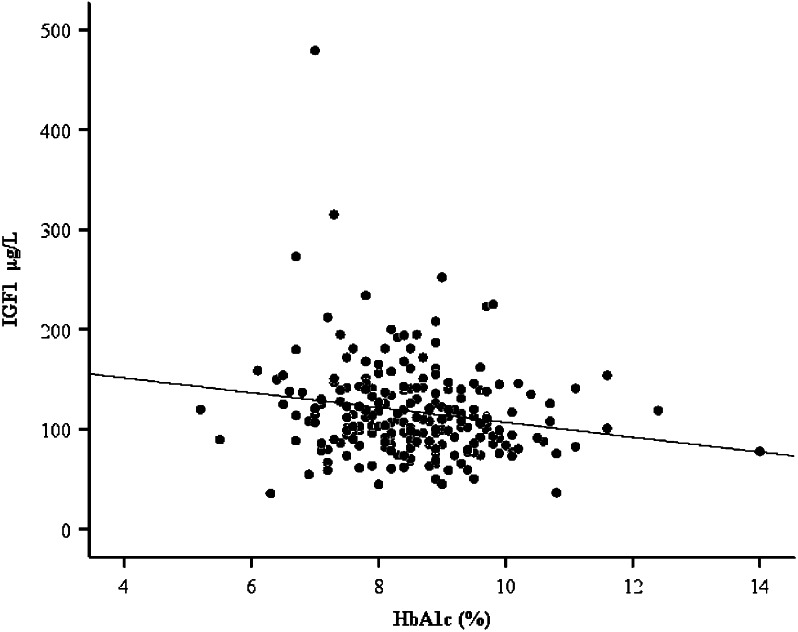
Association between total IGF1 and HbA1c levels in the total population with a regression coefficient of −7.5 μg/l (95% CI −12.04 to −2.97 μg/l) (*P*=0.001), adjusted for the baseline variables such as age, gender, GH, duration of diabetes, HbA1c, BMI, C-peptide level, hypoglycemia awareness class, number of severe hypoglycemic events the previous year, and daily insulin dose as independent variables.

In the multiple regression analysis levels of IGF1 were negatively associated with the HbA1c level (*P*=0.001), with a regression coefficient of −7.5 μg/l (95% CI −12.04 to −2.97 μg/l) in the total population (Fig. 2). This association remained significant when considering men and women separately (men: *P*=0.01, regression coefficient −6.16 μg/l, 95% CI −10.9 to −1.42 μg/l; women: *P*=0.04, regression coefficient −10.35 μg/l, 95% CI −20.15 to −0.55 μg/l).

### IGF1 and age and gender

**Figure 3:**
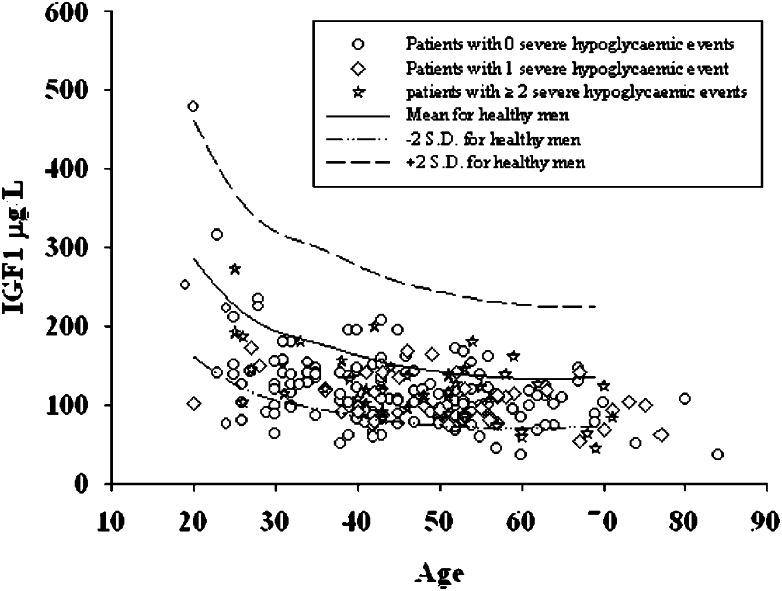
Association between total IGF1 concentration and age in diabetic men with a regression coefficient of −1.26 μg/l, 95% CI −1.71 to −0.81 μg/l) (*P*<0.0001).

As expected, the total IGF1 level was associated with age (*P*<0.0001, regression coefficient −1.26 μg/l, 95% CI −1.71 to −0.81 μg/l; Fig. 3) and was below the level seen in healthy individuals (15). We did not find any association between IGF1 and C-peptide levels (*P*=0.18).

## Discussion

Our data demonstrate that the level of IGF1 is not associated with occurrence of severe hypoglycemia in a population of nonpregnant patients with type 1 diabetes. This contrasts the recent report of an association between low IGF1 and recurrent severe hypoglycemia during pregnancy in women with type 1 diabetes (5). Even when confining the analysis to the female part of the cohort, no association could be detected. The different results between the studies may be explained by the fact that the pregnant women were treated to very low HbA1c levels and were heavily exposed to asymptomatic and mild symptomatic hypoglycemia. In pregnant women with type 1 diabetes, the GH/IGF1 axis is also influenced by placental GH and an increased production of IGF binding proteins (16) as well as a downregulation of IGF1 (17), which may be affected by hypoglycemia in ways that are not fully understood. In theory, this may explain the association between low levels of total IGF1 and risk of hypoglycemia in pregnant patients with type 1 diabetes.

IGF1 levels were generally lower in our population of patients with type 1 diabetes than previously reported for healthy subjects (15). This finding is supported by two previous studies including a total of 255 patients with type 1 diabetes (5, 8). IGF1 levels increase if diabetes control is improved (18). A few minor studies have reported a negative association between HbA1c and circulating IGF1 (6, 8). This was confirmed by our study in which IGF1 was negatively associated with HbA1c.

As low HbA1c level is associated with increased risk of severe hypoglycemia (19, 20, 21) as is the case in this study, the opposite relationship between HbA1c and IGF1 would have been expected if low IGF1 levels in the nonpregnant patients with diabetes predict severe hypoglycemia as in pregnant women with type 1 diabetes (5). Patients in poor metabolic control and consequently high HbA1c levels, however may blunt a potential association between glycemic control and risk of severe hypoglycemia. This may also explain the lack of association between IGF1 levels and severe hypoglycemia.

Surprisingly, we did not find any relationship between IGF1 and C-peptide levels, which in some studies have been suggested to be a major determinant of total IGF1, even more pronounced than glycemic control (8).

The strengths of this study are the large size of the cohort and the thorough characterization of the hypoglycemic phenotype and endpoints. Limitations are the facts that it is a *post hoc* analysis and that samples were collected in nonfasting conditions. Moreover, it is not known whether the women were postmenopausal, on hormonal replacement therapy or using anticonception that could affect the IGF1 level (13, 22).

In conclusion, our study did not find any association between circulating levels of IGF1 and rates of severe, mild symptomatic, or biochemical hypoglycemia. Furthermore, it confirms that circulating IGF1 concentrations are low and negatively associated with glycemic control as indicated by HbA1c in type 1 diabetes. The reason and the effects of this association remain to be established. IGF1 is implicated in both muscle and bone turnover as well as lipid metabolism and growth (23, 24, 25), and our study supports that a low IGF1 level in patients with type 1 diabetes may increase by lowering HbA1c and thereby diminish the negative influence of a low IGF1 on the body.

## Figures and Tables

**Table 1:** Clinical characteristics of 228 patients with type 1 diabetes. Values are mean (s.d.), median (range), or percent when indicated.

	
Age	46 (13)
Gender (females/males) (%)	40/60
BMI (kg/m^2^)	24.9 (3.5)
Age at onset of diabetes (years)	25 (14)
Duration of diabetes (years)	21 (12)
C-peptide (pmol/l) median (range)^a^	16 (0–400)
GH (μg/l) median (range)	1.00 (1–19.7)
HbA1c (%)	8.5 (1.0)
Retinopathy (%)	54
Microalbuminuria (30–300 mg/24 h) (%)	16.2
Macroalbuminuria (>300 mg/24 h) (%)	8.8
Peripheral neuropathy (%)	35
Autonomic neuropathy (%)	9
Hypertension (%)	20
Macrovascular complications (%)	7
≥4 insulin injections per day (%)	84
Daily insulin dose (IU)	50 (19)
Hypoglycemia awareness (normal/impaired/unaware) (%)	40/46/14

a Detection limit 10 pmol/l. A total of 42.7% were below the detection limit.

## References

[1] Juul A, Møller S, Mosfeldt-Laursen E, Rasmussen MH, Scheike T, Pedersen SA, Kastrup KW, Yu H, Mistry J, Rasmussen S, Müller J, Henriksen J, Skakkebaek NE (1998). The acid-labile subunit of human ternary insulin-like growth factor binding protein complex in serum: hepatosplanchnic release, diurnal variation, circulating concentrations in healthy subjects, and diagnostic use in patients with growth hormone deficiency. Journal of Clinical Endocrinology and Metabolism.

[2] Sharp PS, Fallon TJ, Brazier OJ, Sandler L, Joplin GF, Kohner EM (1987). Long-term follow-up of patients who underwent yttrium-90 pituitary implantation for treatment of proliferative diabetic retinopathy. Diabetologia.

[3] Luft R, Olivecrona H, Ikkos D, Kornerup T, Ljunggren H (1955). Hypophysectomy in man; further experiences in severe diabetes mellitus. BMJ.

[4] Bliddal H, Thorsteinsson B, Munkgaard S (1983). Long-term insulin-dependent diabetes mellitus with secondary pituitary insufficiency and regression of retinopathy. Acta Medica Scandinavica.

[5] Ringholm NL, Juul A, Pedersen-Bjergaard U, Thorsteinsson B, Damm P, Mathiesen ER (2008). Lower levels of circulating IGF-I in type 1 diabetic women with frequent severe hypoglycaemia during pregnancy. Diabetic Medicine.

[6] Tan K, Baxter RC (1986). Serum insulin-like growth factor I levels in adult diabetic patients: the effect of age. Journal of Clinical Endocrinology and Metabolism.

[7] Dills DG, Allen C, Palta M, Zaccaro DJ, Klein R, D'Alessio D (1995). Insulin-like growth factor-I is related to glycemic control in children and adolescents with newly diagnosed insulin-dependent diabetes. Journal of Clinical Endocrinology and Metabolism.

[8] Hedman CA, Frystyk J, Lindstrom T, Chen JW, Flyvbjerg A, Orskov H, Arnqvist HJ (2004). Residual beta-cell function more than glycemic control determines abnormalities of the insulin-like growth factor system in type 1 diabetes. Journal of Clinical Endocrinology and Metabolism.

[9] Ekman B, Nystrom F, Arnqvist HJ (2000). Circulating IGF-I concentrations are low and not correlated to glycaemic control in adults with type 1 diabetes. European Journal of Endocrinology.

[10] Pedersen-Bjergaard U, Agerholm-Larsen B, Pramming S, Hougaard P, Thorsteinsson B (2003). Prediction of severe hypoglycaemia by angiotensin-converting enzyme activity and genotype in type 1 diabetes. Diabetologia.

[11] Pedersen-Bjergaard U, Pramming S, Thorsteinsson B (2003). Recall of severe hypoglycaemia and self-estimated state of awareness in type 1 diabetes. Diabetes/Metabolism Research and Reviews.

[12] Hanaire-Broutin H, Sallerin-Caute B, Poncet MF, Tauber M, Bastide R, Chale JJ, Rosenfeld R, Tauber JP (1996). Effect of intraperitoneal insulin delivery on growth hormone binding protein, insulin-like growth factor (IGF)-I, and IGF-binding protein-3 in IDDM. Diabetologia.

[13] Lukanova A, Lundin E, Zeleniuch-Jacquotte A, Muti P, Mure A, Rinaldi S, Dossus L, Micheli A, Arslan A, Lenner P, Shore RE, Krogh V, Koenig KL, Riboli E, Berrino F, Hallmans G, Stattin P, Toniolo P, Kaaks R (2004). Body mass index, circulating levels of sex-steroid hormones, IGF-I and IGF-binding protein-3: a cross-sectional study in healthy women. European Journal of Endocrinology.

[14] Bolli G, de Feo P, Compagnucci P, Cartechini MG, Angeletti G, Santeusanio F, Brunetti P, Gerich JE (1983). Abnormal glucose counterregulation in insulin-dependent diabetes mellitus. Interaction of anti-insulin antibodies and impaired glucagon and epinephrine secretion. Diabetes.

[15] Friedrich N, Krebs A, Nauck M, Wallaschofski H (2010). Age- and gender-specific reference ranges for serum insulin-like growth factor I (IGF-I) and IGF-binding protein-3 concentrations on the Immulite 2500: results of the Study of Health in Pomerania (SHIP). Clinical Chemistry and Laboratory Medicine.

[16] Westwood M (1999). Role of insulin-like growth factor binding protein 1 in human pregnancy. Reviews of Reproduction.

[17] Fuglsang J, Lauszus F, Flyvbjerg A, Ovesen P (2003). Human placental growth hormone, insulin-like growth factor I and -II, and insulin requirements during pregnancy in type 1 diabetes. Journal of Clinical Endocrinology and Metabolism.

[18] Amiel SA, Sherwin RS, Hintz RL, Gertner JM, Press CM, Tamborlane WV (1984). Effect of diabetes and its control on insulin-like growth factors in the young subject with type I diabetes. Diabetes.

[19] Pramming S, Thorsteinsson B, Bendtson I, Binder C (1991). Symptomatic hypoglycaemia in 411 type 1 diabetic patients. Diabetic Medicine.

[20] Allen C, LeCaire T, Palta M, Daniels K, Meredith M, D'Alessio DJ (2001). Risk factors for frequent and severe hypoglycemia in type 1 diabetes. Diabetes Care.

[21] The DCCT Research Group (1997). Hypoglycemia in the Diabetes Control and Complications Trial. The Diabetes Control and Complications Trial Research Group. Diabetes.

[22] Lin CM, Huang YL, Lin ZY (2009). Influence of gender on serum growth hormone, insulin-like growth factor-I and its binding protein-3 during aging. Yonsei Medical Journal.

[23] Colao A, Di Somma C, Filippella M, Rota F, Pivonello R, Orio F, Vitale G, Lombardi G (2004). Insulin-like growth factor-1 deficiency determines increased intima-media thickness at common carotid arteries in adult patients with growth hormone deficiency. Clinical Endocrinology.

[24] Jehle PM, Schulten K, Schulz W, Jehle DR, Stracke S, Manfras B, Boehm BO, Baylink DJ, Mohan S (2003). Serum levels of insulin-like growth factor (IGF)-I and IGF binding protein (IGFBP)-1 to -6 and their relationship to bone metabolism in osteoporosis patients. European Journal of Internal Medicine.

[25] Succurro E, Arturi F, Grembiale A, Iorio F, Laino I, Andreozzi F, Sciacqua A, Hribal ML, Perticone F, Sesti G (2010). Positive association between plasma IGF1 and high-density lipoprotein cholesterol levels in adult nondiabetic subjects. European Journal of Endocrinology.

